# Osteoclasts in Tumor Biology: Metastasis and Epithelial-Mesenchymal-Myeloid Transition

**DOI:** 10.3389/pore.2021.609472

**Published:** 2021-04-30

**Authors:** Kemal Behzatoglu

**Affiliations:** Acibadem Health Group, Pathology Department, Istanbul, Turkey

**Keywords:** tumor biology, metastasis, breast carcinoma metastasis, giant cell tumor of bone, EMMT, osteoclast, epithelial-mesenchymal-myeloid transition

## Abstract

Osteoclast is a specialized cell that originates from monocytic lineage, communicates closely with osteoblasts under physiological conditions, participates in bone modeling and re-modeling, contributes to calcium homeostasis and osteoimmunity. In pathological conditions, it is involved in many tumors such as giant cell bone tumor (osteoclastoma), aneurysmal bone cyst, osteosarcoma, and metastatic cancers, and it usually causes local spread and progression of the tumor, working against the host. Since osteoclasts play an active role in primary bone tumors and bone metastases, the use of anti-osteoclastic agents significantly reduces the mortality and morbidity rates of patients by preventing the progression and local spread of tumors. Osteoclasts also accompany undifferentiated carcinomas of many organs, especially pancreas, thyroid, bladder and ovary. Undifferentiated carcinomas rich in osteoclasts have osteoclastoma-like histology. In these organs, osteoclastoma-like histology may accompany epithelial carcinomas, and *de novo*, benign and borderline tumors. Mature and immature myeloid cells, including osteoclasts, play an active role in the tumor progression in primary and metastatic tumor microenvironment, in epithelial-mesenchymal transition (EMT), mesenchymal-epithelial-transition (MET), and cancer stem cell formation. Additionally, they are the most suitable candidates for cancer cells in cell fusion due to their evolutionary fusion capabilities. Myeloid features and markers (CD163, CD33, CD68 etc.) can be seen in metastatic cancer cells. Consequently, they provide metastatic cancer cells with motility, margination, transmigration, chemotaxis, phagocytosis, angiogenesis, matrix degradation, and resistance to chemotherapy. For these reasons, we think that the concept of Epithelial-Mesencyhmal-Myeloid-Transition (EMMT) will be more accurate than EMT for cancer cells with myeloid properties.

## Introduction

The development of target-specific therapies in tumor biology enables us to access new data about the behavior and microenvironment of tumors. Studies on the role of osteoclasts (OCLs) in tumor biology have accelerated in recent years, and OCL inhibitors have been successfully used to treat metastatic cancers, especially breast cancer, multiple myeloma, and various diseases such as osteosarcoma, giant cell bone tumor, and aneurysmal bone cyst. OCLs are one of the most important cells in the tumor microenvironment, especially in bone metastases. They also cause complications associated with high morbidity and mortality, such as hypercalcemia, fracture, and bone resorption, in addition to expressing molecules leading to tumor progression [1–4]. The inhibition of OCLs in addition to tumor treatment significantly contributes to patient recovery and increases both their life span and quality of life [1, 2].

OCLs are specialized cells that are derived from the monocytic lineage. They participate in bone modeling and remodeling, and are involved in calcium homeostasis and hematopoiesis modulation, bone healing, and osteoimmunity under physiological conditions [4–7]. OCLs work with osteoblasts to carry out these tasks, especially in bone remodeling, and contribute to the regulation of osteoblasts. This dependence on physiological conditions means that OCLs are also influenced by environmental effects (the microenvironment). The normal regulation of OCLs by osteoblasts under physiological conditions is more complex due to the involvement of B lymphocytes, T lymphocytes, natural killer (NK) cells, monocytes, macrophages, dendritic cells, and tumor cells in the presence of inflammation and tumors. OCLs often play a role in tumor progression and inflammation due to these complex relationships. They can lead to significant complications such as bone and cartilage erosion in inflammatory diseases such as rheumatoid arthritis, in addition to tumor progression, metastasis, bone fractures, and hypercalcemia.

In this review, we will first investigate the nature and physiological features of OCLs and discuss the two common prototype tumors that accompany these cells: giant cell tumor of the bone (GCTB) and metastatic breast cancer in the bone. We will also focus on the histological characteristics, nature, and nomenclature of GCTB-like tumors rich in localized OCLs in parenchymatous organs, as these are relatively less common and have not been thoroughly studied. In this article, we discuss and propose for the first time the concept of Epithelial-Mesenchymal-Myeloid Transition (EMMT), which we believe is important in cancer progression.

### Osteoclasts Function

As living beings made the water-to-land transition during evolution, the cells of the bones and immune system in the vertebrae may have had to develop synchronously in order to protect the premature cells of the immune system from UV rays and to meet the calcium needs of the body from internal resources. This has resulted in a close interaction between bones and immune system cells [8–10]. Furthermore, there is a close relationship between cells of these systems and cytokines, chemokines, transcription factors, and signaling molecules. The best examples are the development of receptor activator of nuclear factor-κB ligand (RANKL), which is important for osteoclastogenesis, and the receptor activator of nuclear factor-κB (RANK), which is involved in the development of the immune system (thymic medulla, lymph nodes, and microvillus cells in the intestine) [11].

OCLs have many common characteristics with monocytes, dendritic cells, and granulocytes, as they are derived from the same monocytic lineage. As they may be the final element of this sequence during evolution, OCLs cannot transform into monocytes or dendritic cells under physiological or pathological conditions, but these cells can easily transform into OCLs with appropriate stimulation under pathological conditions [11–14]. The OCL pool is therefore large and has high heterogeneity and spasticity. Although the most important function of OCLs is their role in bone resorption, they also phagocytize the waste material released during this process via endocytosis and micropinocytosis. In this sense, we can also consider OCLs to be the largest macrophage in the body. Multinucleation for bone resorption is similar to the process by which macrophages can transform into Langhans cells, foreign body giant cells, and Touton-type giant cells. OCLs also have the capacity to regulate T cell activation to ensure effective antigen presentation and processing, as well as activating the T-cell response in an antigen-dependent manner. This process is also observed during phagocytosis in other cells with a monocytic lineage, such as monocytes, macrophages, and dendritic cells [4]. OCLs can act in the direction of immune suppression or inflammation, depending on the effect of the cell from which they develop and their microenvironment. They protect tumor cells from lymphocytes by inhibiting the T-cell-mediated cytotoxicity of CD4^+^ and CD8^+^ cells in malignancies such as multiple myeloma and breast cancer metastases [15, 16].

To understand the functions of OCLs in physiological and pathological environments, it is important to elucidate the bone remodeling process, which is a typical feature of the osteoclastogenesis mechanism. Ensuring bone homeostasis via the interaction between osteoblasts and OCLs is the first step in bone degradation, followed by new bone building. The two most important molecules for the differentiation, activation, and survival of OCLs under physiological conditions are macrophage-colony stimulating factor (M-CSF) and RANKL, which are expressed by osteoblasts. The M-CSF molecule expressed by osteoblasts and bone marrow stromal cells is effective on monocytes*/*macrophages*/*preosteoclasts and supports the differentiation of preosteoclasts toward osteoclastogenesis, with an increase in the RANK receptor. RANKL is an important molecule in the development of the immune system and plays an essential role in osteoclastogenesis [6, 8, 12]. RANKL ensures the activation and survival of OCLs by binding to the RANK receptor on their surface. Osteoprogenitor (OPG) molecules, which are also expressed in osteoblasts, aid in homeostasis by directing OCLs to apoptosis by binding to RANKL, thus ending bone resorption. The RANKL/RANK/OPG axis is the fundamental unit of osteoclastogenesis.

The relationship between OCLs and osteoblasts in the bone remodeling process is similar between tumor cells and OCLs in metastases, neoplastic stromal cells, and OCLs in giant-cell tumor of the bone (GCTB), and between malignant osteoblastic cells and OCLs in osteosarcoma. In addition to RANKL, RANK, and OPG expressed by osteoblasts and OCLs, many molecules such as interleukin (IL)-1, IL-6, IL-7, IL-17, IL-23, IL-33, IL-34, IL-35, tumor necrosis factor α (TNF-α), interferon γ (IFNγ), transforming growth factor β (TGFβ), prostaglandin E2 (PGE2), parathyroid hormone-related protein (PTHrP), intercellular adhesion molecule 1 (ICAM1), platelet-derived growth factor (PDGF), macrophage inflammatory protein 1A (MIP1a), bone morphogenetic proteins (BMPs), and fibroblastic growth factor (FGFs) are released during bone resorption. These molecules may be expressed by primary or metastatic tumor cells or by various cells such as T cells, CD4 T cells, Th17 Cells, CD4 Treg cells, CD8 T cells, CD8 Treg cells, NK T cells, γ∞T cells, B cells, dendritic cells, monocytes*/*macrophages, or neutrophils, which are present in the environment under physiological and pathological conditions. These molecules also play a role on osteoclastogenesis [4–8, 17–28]. While most of these molecules increase osteoclastogenesis, other cells and molecules such as IFNγ, IL-4, IL-10, IL-12, IL-18, and regulatory T cells (Treg), are effective in inhibiting osteoclastogenesis [5, 6, 28].

## The Role of Osteoclasts in the Bone in Metastatic Breast Cancer

**FIGURE 1:**
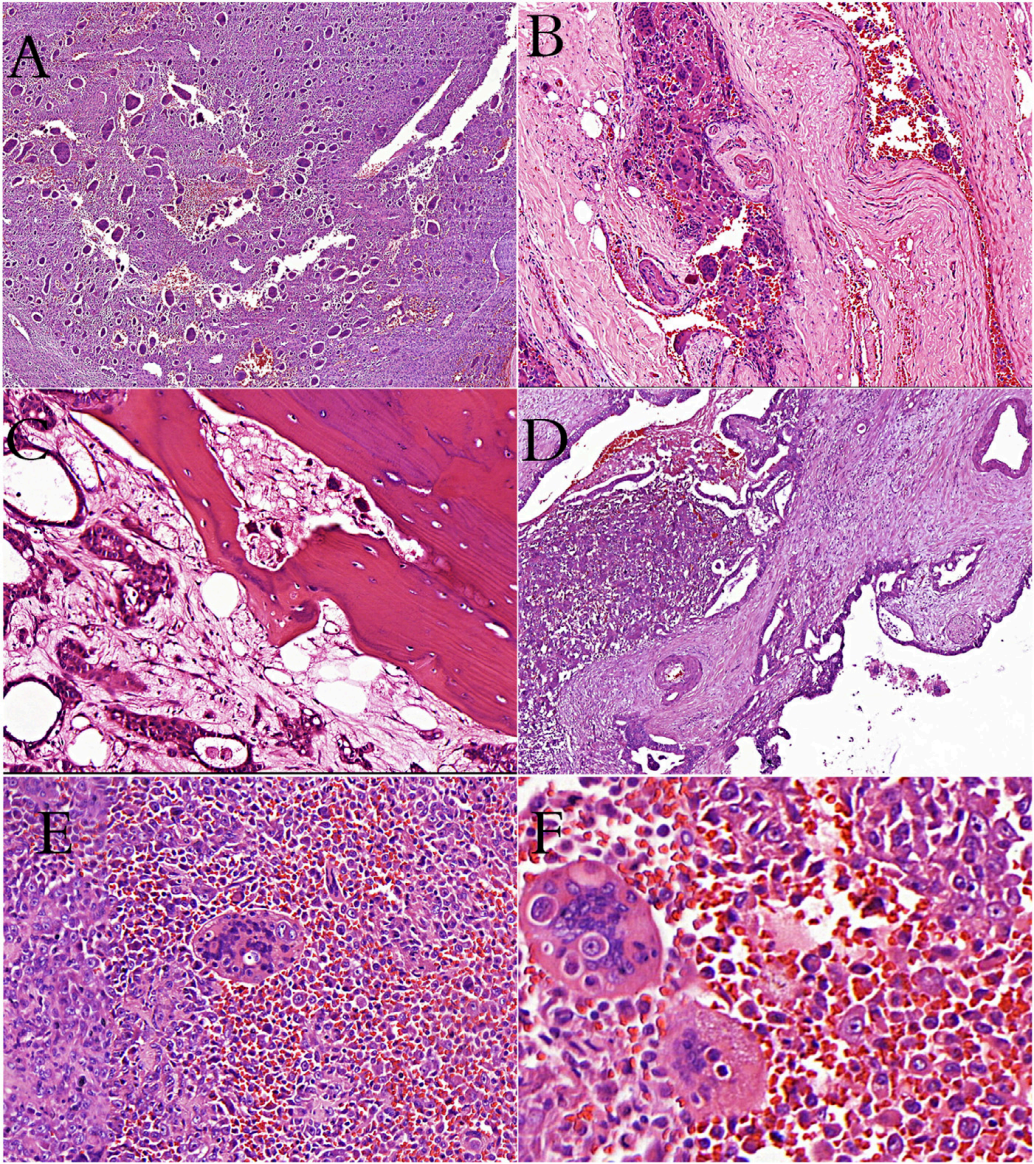
**(A)**: Giant-cell tumor of bone showing the characteristic giant cells and mononuclear cells (H&E stain). **(B)**: Giant-cell tumor of bone showing vascular invasion (H&E stain). **(C)**: Metastatic breast carcinoma cells and osteoclast-like cells in bone tissue (H&E stain). **(D)**: İntraductal polypoid growth of osteoclast-rich undifferentiated carcinoma in pancreas (H&E stain). **(E, F)**: Cells of thyroid undifferentiated carcinoma in osteoclastic cells (phagocytosis? or fusion?).

Breast cancer is one of the tumors that most commonly metastasize to the bone, constituting the vast majority of metastases wherein osteoclastogenesis is predominantly accompanied by osteolysis and bone resorption (Figure 1C). The prevalence of bone metastasis in breast cancer is approximately 65–75% [29]. Bone metastases are important in these patients due to complications such as pain, spinal cord compression fracture, bone marrow aplasia, and potentially fatal hypercalcemia [30].

Although the preference of breast cancer metastases for the bone is consistent with the “Seed and Soil” theory of Paget in 1889, the “circulating theory” suggested by Ewing in 1928 is also appropriate [31, 32]. As a result, cancer cells that enter the vessels can move to many organs, but only find a suitable environment to live and grow in some of them. It is believed that the primary tumor prepares the area for future metastasis by expressing several growth factors and cytokines, such as IL-6, IL-10, VEGFr, and bone matrix proteins [16, 33–35]. Rucci and Teti have emphasized that collective expression and genetic similarities with the organ cells are important for tumor cells metastasizing to the bone to proliferate, and designated these conditions as “osteomimicry” [36]. Osteomimicry cells correspond to osteoblasts in metastatic cancers; indeed, it is the osteoblasts that manage osteoclastogenesis and secrete the appropriate molecules, as seen under physiological conditions. Breast cancer cells replace osteoblasts in the metastatic area and use this cycle for their growth and survival. There are also many features of OCLs that are similar to tumor cells. The RANKL/RANK pathway, which is very important in osteoclastogenesis and immune system development, is also effective in breast organogenesis and causes progression of primary breast cancer [37–41]. Breast cancer cells, immune system cells, osteoblasts, and OCLs in the RANKL/RANK metastatic field microenvironment interact with each other due to the similarities in their regulation and create a cycle favoring cancer growth. Tumor cells directly stimulate osteoclastogenesis by expressing molecules such as RANKL, IL-6, IL-8, IL-11, IL-1, and IL-1B; they also indirectly stimulate osteoclastogenesis by increasing RANKL production in osteoblasts via PTHrP expression [26, 27, 42]. PTHrP also supports the osteolytic cycle by lowering the level of OPG, an osteoclastogenesis inhibitor [40]. Growth factors such as TGFβ, FGFs, IGFs, BMPs, PDGF, and Ca^++^ molecules, which are stored in the bone matrix during bone resorption by OCLs, stimulate cancer growth [43, 44].

TGFβ, found in the metastatic microenvironment in bone, increases the histological and molecular differentiation of tumor cells with epithelioid features called epithelial–mesenchymal transition (EMT) cells into mesenchymal cells [39, 40]. These mesenchymal cells have high mobility, are more aggressive, show more metastatic capacity, and are more resistant to chemotherapy. The RANK level in the primary breast tumor area is directly proportional to the increase in EMT cells [43]. The widespread RANK in the metastatic microenvironment may also induce EMT transformation. In addition, the immunosuppressive effect of OCLs on this cycle facilitates the growth and spread of tumor cells. The inhibition of OCLs, which work almost entirely in favor of the metastatic tumor in this cycle, seems to be very important in treatment. Indeed, drugs that inhibit osteoclastogenesis at various levels, especially denasumab, are used in treatment regimens by increasing the survival of patients, reducing complications, and increasing the quality of life [44]. In addition, RANKL/RANK inhibitors such as denasumab can increase the effectiveness of immunotherapy (checkpoint blockade) [45].

In addition to EMT, some researchers have recently proposed the concept of epithelial–myeloid transition (EMyeT) [46, 47]. Reportedly, several molecules on myeloid cells that are present normally and during their movement to areas of inflammation in the body, such as margination, transmigration, chemotaxis, and phagocytosis, are also present on cancer cells. Furthermore, these cells can make use of or gain these features to reach the primary site or the intravascular and metastatic areas. In light of this observation, studies suggest that the term EMyeT may be a more accurate definition of this process. Those who support the EMyeT concept emphasize that mesenchymal molecules such as those involved in spindle formation (fibroblast-like) are also the molecules found during inflammation in myeloid series cells (monocytes, dendritic cells, OCLs, and granulocytes). These molecules include Snail, ZEB, Twist1, vimentin, N-cadherin, and fibronectin, which tumor cells use to pass intravascularly from the primary area and for their intravascular survival. In addition, studies state that this hypothesis is supported by the expression of M-CSF, which stimulates the myeloid series, and RANKL/RANK, which contributes to bone remodeling in cancer progression, especially in breast cancer.

The EMyeT concept appears to be the merged and expanded version of the abovementioned “seed and soil” and “osteomimicry” hypotheses, and is consistent with the role of OCLs in metastasis emphasized in this article. The problem with the EMyeT hypothesis is deciding whether it should be used instead of EMT. We propose that we should consider these two hypotheses not as opposing hypotheses, but rather as complementary concepts. Mesenchymal cells (fibroblasts, osteoblasts, chondroblasts, lipoblasts, etc.) and myeloid cells (monocytes, dendritic cells, OCLs, and granulocytes) originate from the mesoderm and are derived from stem cells. Therefore, they share many histological and genetic features. The two concepts can be used separately, but the term EMMT appears be more appropriate in this context.

## Osteoclasts in Giant Cell Tumor of the Bone

OCLs are associated with many tumors of the bone, such as GCTB, osteosarcoma, aneurysmal bone cyst, metaphyseal fibrous defect, and chondroblastoma, and possibly contribute to the lytic features of these tumors. Although OCLs accompany many bone tumors, we will focus on GCTB; in this tumor type, OCLs are large (multinucleated) and very high in number, giving the tumor its name (osteoclastoma), and have an essential role in its pathogenesis.

OCLs are the most prominent feature of GCTB, although they do not compose the neoplastic component of the tumor. GCTB is a benign, locally aggressive neoplasm with an unclear nature. It may have frequent recurrences and is occasionally capable of benign metastasis. It accounts for approximately 6% of all bone tumors and is usually located in the epiphysis of long bones, but can also invade the metaphysis and joint space [48–50]. The histological appearance consists of spindle-shaped cells (stromal cells) that form the neoplastic component in a hypervascular background, mononuclear monocytic cells that form the reactive component, and osteoclastic giant cells (Figures 1A,B). The prominence of osteoclastogenesis in this tumor causes large tumor diameter and local aggressiveness due to marked bone resorption in the tumor. In terms of genetic alterations in this tumor, the 3.3 histone mutation of the H3F3A gene has recently been described [51]. GCTB is generally accepted as a neoplastic process, but it has also been suggested that the neoplastic stromal cells could be reactive [52].

The RANKL*/*RANK/OPG axis works in favor of osteoclastogenesis in GCTB, as in metastatic breast cancers. Although OPG is also expressed in stromal cells, it cannot compete with the presence of high levels of RANKL in the microenvironment. The OCLs in GCTB are composed of monocytes originating from the same lineage rather than from preosteoclastic cells [53, 54]. The presence of so many monocytes in the tumor is thought to be the result of macrophage chemotaxis caused by the expression of many cytokines, such as IL6, IL-11, IL-7, PTHrP, and TGFβ from neoplastic stromal cells [53, 54]. TGFβ also plays a role in the presence of molecules such as TNF-α and IL-1, which are found in the tumor microenvironment and mainly increase osteoclastogenesis. Monocytes that can transform into OCLs under pathological conditions carry many receptors and ligands that are similar to OCLs. Stromal cells act like osteoblasts and express M-CSF, RANKL, and IL-34, and induce osteoclastogenesis from monocytes through RANK receptors on the surface of monocytes [14]. RANKL and RANK express nuclear factor of activated T cell c1 (NFATc1) in the cell after binding, while NFATc1 ensures the transcription of several factors (cathepsin K, TRAP, calcitonin receptor, and β3 integrin) that are essential for OCLs [54]. NFATc1 also supports osteoclastogenesis by increasing monocyte fusion through increased osteoclast stimulatory transmembrane protein (OC-STAMP) expression on monocytes [55]. Another protein that increases RANKL expression is parathyroid hormone type 1 protein (PTH1P), which is expressed in stromal cells [56]. The PTH1 receptor is found on osteoclastic giant cells and not on normal OCLs, and may be one of the reasons for the histological similarity between GCTB and brown tumors [48, 56].

An important feature of GCTB is that the osteoclastic cells are larger multinucleated cells than normal OCLs. There may be many reasons for this, such as: the development of giant cells from monocyte*/*macrophage mononuclear cells originating from the same lineage as OCLs rather than preosteoclastic mononuclear cells; the increased expression of osteoclastogenic cytokines and chemokines such as IL-6, IL-11, IL-17, IL-34, and TGFβ; and the presence of PTH1R in OCLs and consequent RANKL and RANK overexpression [53, 54]. A study has reported that stromal neoplastic cells could be transformed into osteoclastic giant cells by fusion in spine-located GCTB, but further studies are required [57]. Although GCTB is a locally aggressive and benign tumor, the frequency of vascular invasion, the abundance of OCLs in the intravascular thrombus, and the occasional presence of OCLs alone in the vessel suggest that OCLs may play a role in metastasis. OCL origin in GCTB is mostly monocytic, and these cells are generally found in the intravascular or perivascular area, also suggesting a role in metastasis.

The use of anti-osteoclastic agents such as denosumab has recently become common for treating many bone tumors wherein osteoclastogenesis is important in their pathology, such as GCTB, osteosarcoma, and aneurysmal bone cysts, and positive results have been reported [44]. In particular, the OCL inhibitor denosumab, which is an antibody that targets RANKL, is used in cases of GCTB that are unresectable and/or pose a risk for the joints or with their localization [58]. The most important histological changes after treatment are as follows: a significant decrease in OCLs, a smaller decrease in neoplastic stromal cells, increased spindling, decreased mitotic index, and the appearance of new bone areas in various patterns depending on treatment duration [59–63]. GCTB can have a histological appearance similar to that of fibrous dysplasia, non-ossifying fibroma, and even osteosarcoma depending on bone production and the duration of denosumab use [62, 63]). The abovementioned histological changes due to denosumab usage also provide new perspectives on the nature of GCTB. Stromal neoplastic cells in GCTB are mainly considered to be undifferentiated with an indeterminate nature. However, these cells contain early stage osteogenic markers, are positive for the immunohistochemical osteogenic marker SAT2, and sometimes contain osteoid. This evidence supports the notion of osteoblastic differentiation of these cells. Stromal cells are thought to mainly have a bone lining cell phenotype [52]. The prominence of osteogenic features after denosumab treatment suggests that osteoblastic features are suppressed in GCTB. OCLs are also known to have the ability to suppress osteoblastic differentiation [64]. In addition, many molecules that suppress osteoblastogenesis within the GCTB microenvironment, such as IL-3, IL-7, IL-6, TNF-α, semathorin 4D, and plexin-B1, may also be responsible [65–67]. There are some reports that stromal cells can show adipocytic and chondrocytic differentiation as well as osteogenic differentiation [68, 69].

The effects of OCLs in a primary tumor such as GCTB localized in bone tissue, and in secondary tumors such as bone metastasis of breast cancer, can be summarized as follows: they open up space for the tumor by causing bone resorption and help its spread via invasion in the bone and soft tissue; they affect T cells, lead to immunosuppression, and help the tumor grow and spread; some molecules that emerge during bone resorption cause tumor growth and progression; they support changes related to EMT, EMyeT, and EMMT, which make tumor cells more aggressive and increase their metastatic capacity; and they possibly contribute to vascular invasion in GCTB.

## Giant Cell Tumor of the Bone-Like Histology in Parenchymatous Organs (*de novo* or Accompanied Benign, Borderline/Low-Grade Epithelial Tumors, and Invasive Carcinomas)

Although GCTB is a primary tumor of the bone, it can also be localized in soft tissue with similar clinical, histological, and molecular features. The tumor is called a “soft tissue giant cell tumor of low malignant potential” in soft tissue, and is a known entity that presents no obvious diagnostic problems [70, 71]. However, tumors with GCTB-like histology are also seen in several parencymatous organs such as the ovary, urinary bladder, breast, extrabiliary system, and especially the pancreas [72–76]. Although they are seen as “*de novo*” in the parenchymatous organs, the tumors are mostly accompanied by benign epithelial, borderline/low-grade, or invasive carcinomas of these organs, which causes challenges regarding their diagnosis, nature, and nomenclature. Aside from their GCTB-like histology in the parenchymatous organs, the tumors can also present as anaplastic carcinoma or sarcomatous carcinoma. Here, we will focus more on lesions with a GCTB-like histology. We will also discuss GCTB-like tumors accompanying ovarian, pancreatic, and retroperitoneal cystic mucinous neoplasms, together with the mural nodule concept, as the nature of mucinous tumors is still unclear.

“De novo” GCTB-like lesions show clinical, histological, and immunohistochemical characteristics (epithelial markers are generally negative) similar to those of primary GCTB, and surgical resection is generally curative [75, 77–81]. Although very rare, malignant GCTB-like tumors with no epithelial differentiation have been reported, and the prognosis of these cases is poor [80]. Some GCTB-like tumors have epulis-like histology and mainly appear as reactive lesions. Mononuclear cells show positive staining with mesenchymal and myeloid markers such as CD68, vimentin, LCA, SMA, and S-100, in varying proportions.

Osteoclastic cells show their classical immunohistochemical features; they are generally CD68-positive but negative for epithelial markers, and show a low mitotic index and low Ki-67 proliferation index. Rarely, cases with mostly malignant characteristics that are not accompanied by any neoplasm and which are positive for epithelial markers (EMA, keratins) have been reported [82–87].

Tumors with GCTB-like histology, other than *de novo* cases, may also accompany benign epithelial, low-grade, and invasive carcinomas of many organs. In general, epithelial and GCTB-like tumors present in an intertwined or overlapping manner or as separate nodules. Tumors are generally evaluated according to the parenchymatous organ*/*system in which they are observed, and are given various names such as “osteoclast-rich undifferentiated carcinoma” [81], “undifferentiated carcinoma with osteoclast” [82], “sarcoma-like mural nodule” [73], and “osteoclastic variant of anaplastic thyroid carcinoma” [84] despite their similarity. The reason for using the expression of “undifferentiated carcinoma” in some tumors with GCTB-like histology accompanying parenchymatous organ tumors is the presence of varying degrees of positivity for epithelial markers and/or additional diagnostic markers, in addition to myeloid-mesenchymal markers.

GCTB-like tumors accompanying epithelial tumors have been reported in low numbers and generally as individual cases in almost every organ, with the pancreas being the organ most commonly involved [82, 84]. A small number of relatively large series on the bladder have also been published [81, 85]. Giant cells (OCLs) in GCTB-like tumors accompanying epithelial tumors are generally positive for at least two osteoclastic markers (CD68, CD54, CD51, TRAP, LCA, and vimentin) [73, 80, 85–87]. Myeloid-mesenchymal markers (such as CD68, CD163, HAM56, HHF35, vimentin, SMA, and S-100) are generally positive in mononuclear cells [73, 80, 85–87], which are considered to be neoplastic, but positive results for epithelial markers (EMA, keratins) are also observed in some cases, demonstrating a mystery cell [73, 80, 85, 86]. The presence of early embryological neuro-muscular cell lineage markers such as nestin, SOX2, and CD56 as well as epithelial, mesenchymal, and myeloid markers, has been reported in osteoclast-rich undifferentiated carcinoma in the bladder, indicating that these cells may also have stem cell features [86]. More studies on stem cell markers (such as CD133 and nepsin) in GCTB-like tumors are needed. In our opinion, most of these tumors are likely to have been positive for these markers.

The positivity of mononuclear cells only for myeloid-mesenchymal markers in some tumors and both epithelial and myeloid-mesenchymal markers in others has led to controversy about the nature of these cells. Regarding these contrasting results, some authors have suggested a mesenchymal origin, while others have suggested an epithelial origin [71, 80, 87–90]. The fact that mononuclear cells in some GCTB-like tumors in the pancreas are positive for the KRAS mutation, which plays a role in the pathogenesis of pancreatic adenocarcinomas, supports the hypothesis that these tumors are of epithelial origin [82, 84]. The nature of GCTB is still uncertain, although positivity for SATB2 observed in some cases suggests an osteoblastic origin due to early osteoblastic markers. This makes the nature of GCTB-like tumors seen in parenchymatous tissues more complex. The positivity of common myeloid-mesenchymal markers as well as epithelial markers in the mononuclear cells, which are considered to be neoplastic, makes it difficult to determine the nature of these tumors. Although osteoid and osteochondroid production in GCTB-like tumors in the parenchymatous organs have been reported in some cases [91–93], the osteoblastic differentiation status of mononuclear cells is unknown, and no studies have been conducted on this subject. GCTB-like tumors may be seen as a differentiated version of carcinoma in invasive tumors, but it is interesting to note that GCTB-like tumors also accompany benign and low-grade epithelial tumors, which requires further investigation. Some authors have reported that molecules such as growth factors expressed from tumors, especially in primary low-grade urothelial tumors in the bladder, may induce GCTB-like tumors in stromal and stem cells [87, 88]. They also emphasized that some of these may be reactive in nature, and that they do not recur after surgical resection [87]. An intraductal/intracystic pattern and even polypoid growth toward the normal ducts are also remarkable in GCTB-like neoplasms of the pancreas [82, 84, 94]. This pattern may mimic intravascular tumor spread, which is common in primary GCTB (Figure 1D). Interestingly, angiolymphatic vascular invasion and the incidence of tumor thrombi in GCTB-like tumors were found to be quite high in one study [82].

We have mentioned the EMyeT concept in breast cancers metastasizing to the bone, and we emphasized that this hypothesis could be valid for tumor cell proliferation at the primary and metastatic sites as well as intravascular spread of tumor cells. However, we also mentioned that EMMT might be a more appropriate term for this phenomenon. There are studies emphasizing that EMT is effective in GCTB-like tumors accompanying epithelial neoplasms seen in the parenchymatous organs [86, 89]. However, some authors have suggested that explaining these lesions with EMT is insufficient, especially in tumors of the pancreas [82]. The positivity of both mesenchymal (vimentin, S-100, SMA, HHF35) and myeloid (CD68, CD163, HAM56, LCA) markers in cases where epithelial markers (EMA, keratins) are positive in mononuclear cells in GCTB-like tumors suggests that the differentiation of these cells can be better explained by the EMMT concept rather than the EMyeT concept. The concepts of EMT, EMyeT, and EMMT that we have discussed herein can explain why tumor cells in other organs have the capacity to be more aggressive, show progression, have increased metastatic activity, and exist in the metastatic area. However, it is difficult to say the same for GCTB-like tumors, as the fact that GCTB-like tumors have a worse prognosis than the conventional carcinomas of the organ in which they are localized is controversial. The prognosis of undifferentiated carcinomas (including GCTB-like, anaplastic carcinoma, and sarcomatoid type) has been emphasized to be better than conventional pancreatic carcinomas in studies with large series [82, 84]. Furthermore, the prognosis has been reported to be better for *de novo* GCTB-like cases and cases where a smaller ductal component of pancreatic adenocarcinoma is observed [82].

The prognosis of these tumors is better than that of conventional ductal carcinomas of the pancreas. We believe that this is the result of evaluating undifferentiated carcinomas together with GCTB-like, anaplastic, and sarcomatoid types, and accompanying *de novo*, epithelial benign and borderline lesions, and invasive carcinomas in the same group. Such results may have been achieved due to the better prognosis of *de novo*, benign, and borderline/low grade epithelial tumors. Different results can be obtained if invasive ductal carcinomas are compared with only undifferentiated cases accompanying ductal carcinomas. A good example of this is thyroid anaplastic carcinoma. Thyroid anaplastic carcinomas show osteoclastic giant cells and sarcomatoid histology. They arise as anaplastic transformation of differentiated thyroid carcinoma (papillary, follicular, or Hürthle cell carcinoma). Most cases have a core of conserved mutations in well-differentiated and anaplastic areas, and mutation rates increase in anaplastic areas [95]. We suggest that this highly aggressive tumor is similar to undifferentiated carcinomas that accompany high-grade cancers in the pancreas and urinary bladder. These tumors with a carcinoma background should be considered undifferentiated, and we believe that EMMT and stem cells are very effective in this process. In another study, it was concluded that stemness induced by EMT plays an important role in anaplastic thyroid carcinoma [96]. In our opinion, “undifferentiated thyroid carcinoma” explains the nature of these tumors better than “thyroid anaplastic carcinoma”; similarly, “undifferentiated carcinoma” provides a better description than metaplastic carcinoma of the breast. It was also emphasized that OCLs are important in invading surrounding cartilage tissues, such as the trachea in thyroid undifferentiated carcinomas [97]. However, it should be noted that GCTB-like lesions accompanying *de novo*, benign, and low-grade epithelial tumors may also be reactive or have good prognosis. This idea also applies to GCTB-like tumors that accompany mucinous neoplasia, which are described below.

## Mucinous Neoplasms and GCTB-Like Nodule/Mural Nodule

Mucinous neoplasms are mainly present in the ovaries and rarely in the pancreas, liver, and retroperitoneum in the form of benign, borderline, and invasive carcinomas. Mucinous neoplasms are rarely accompanied by mural nodules with a GCTB-like morphology [98–102]. The mural nodule may be malignant (anaplastic carcinoma) or can present as a benign nodule with GCTB- or epulis-like histology (also called a sarcoma-like nodule).

Interestingly, mural nodules accompany gastrointestinal-type mucinous neoplasia in the ovary rather than endometrioid tumors of Müllerian origin, clear cell carcinoma, and serous tumors. The nature of mucinous neoplasms remains controversial [103]. The fact that they frequently accompany mature cystic teratomas, their common coexistence with Brenner tumors, and the development of cases with Brenner tumor coexistence from the same monoclonal origin excludes a Müllerian phenotype [103, 104]. In addition, the common coexistence of mucinous neoplasm and Brenner tumor with paraovarian and paratubal Walthard cell nests of benign transitional-type epithelial origin also excludes a Müllerian origin [104].

The origin of pancreatic, retroperitoneal mucinous, and hepatic neoplasms is still controversial, although they show histological and genetic similarity to ovarian mucinous neoplasms. The presence of ovarian-like stroma accompanying pancreatic, retroperitoneal, and hepatic mucinous neoplasms, the lack of a connection between pancreatic mucinous neoplasms and the pancreatic ductal system, localization in the pancreatic neck and tail closer to the midline instead of the pancreatic head, left lobe in the liver, and their more common occurrence in women suggests that they have a teratoid or ovarian remnant nature. The fact that a tumor with such a controversial nature is accompanied by nodules with GCTB-like*/*epulis-like histology, again with an unclear nature, creates a very complex situation regarding its histogenesis. GCTB-like (sarcoma-like) nodules have a benign character and are usually accompanied by benign and borderline mucinous neoplasms. It is therefore suggested that these may be reactive lesions rather than tumors. The problem regarding the nature of the lesion in GCTB-like tumors of other organs is also valid here. However, mucinous neoplasms are also accompanied by malignant anaplastic and sarcomatoid mural nodules that do not include OCLs.

In summary, the well-recognized functions and the important role of OCLs in bone-localized tumors are not fully known in GCTB-like tumors localized in the parenchymatous organs. The absence of bone and cartilage tissue in the environment, as well as the limited knowledge on the nature of the tumor cells and their relationship with OCLs, make it difficult to determine their role in these organs. The widespread invasion and aggressive course of GCTB-like tumors in organs such as the bladder and thyroid suggest that this may be due to the effect of OCLs. We also believe that they have a role in widespread vascular invasions and invasion of the surrounding bone and cartilage tissues in the thyroid. Although the positive effects of OCL inhibitors on prognosis in aggressive cases of GCTB-like undifferentiated carcinoma are not shown, they can be used alongside other treatments. In addition, OCLs in the tumor microenvironment may be involved in the EMMT process of tumor cells in metastatic cancers and osteoclast-rich undifferentiated carcinomas.

## Osteoclast, Other Myeloid Cells and EMMT Concept

Myeloid cells are among the important cell groups belonging to the hemopoietic system of our body, which are involved in primary and secondary wound healing, antigen processing and presentation, cytokine synthesis, phagocytosis, and giant cell (OCL, Langhans, etc.) formation, which is one of the best examples of cell fusion. They are highly mobile cells that have properties such as nesting-migration, adhesion-transmigration, and chemotaxis-activation on their way to the target organ [105]. These features are the basic characteristics that cancer cells desire to have, especially during metastasis. Myeloid cells, especially mature macrophages, play a role in the immune response to tumors; however, a separate group of cells called myeloid-derived suppressor cells (MDSCs), which are defined as immature myeloid cells that develop from hematopoietic stem cells in the bone marrow and suppress the immune response to the tumor, has also been defined [16, 106]. These MDSCs (commonly express the myeloid marker CD33) are immature myeloid cells that work in favor of the tumor by suppressing T cells and NKs in the microenvironment of primary tumors and during bone metastasis. MDSCs can differentiate into tumor-associated macrophages (TAMs), tumor-associated dendritic cells (TADCs), and tumor-associated granulocytes (TAGs) that support tumor cell proliferation and invasion [16, 107]. Apart from immune suppression, MDSCs play an important role in tumorigenesis, with many effects on tumor metastasis, migration to the tumor site or pre-metastatic niche, angiogenesis, stem cell-like features in tumor cells, EMT, and development of MET in the metastatic focus [16, 108]. Taking these features into consideration, MDSCs play an active role in bone metastasis.

**FIGURE 2:**
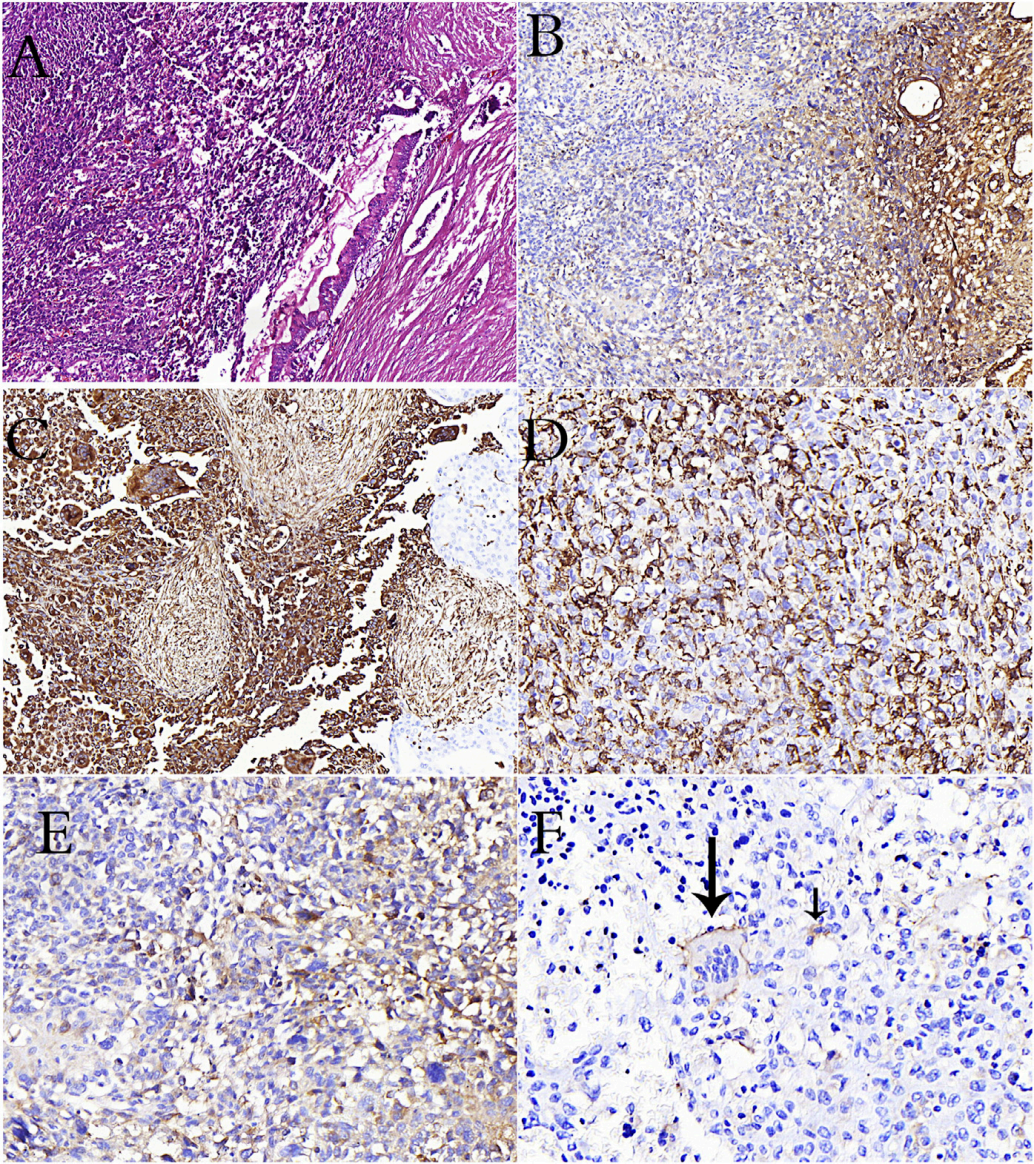
**(A)**: PanIN in the duct adjacent to undifferentiated carcinoma rich in osteoclasts in the pancreas (H&E stain). **(B)**: Loss of e-cadherin in undifferentiated carcinoma in immunohistochemical study **(C)**: Positivity of vimentin in osteoclast-rich undifferentiated carcinoma cells in immunohistochemical study **(D)**: CD68 positivity is remarkable in osteoclastic cells and undifferentiated carcinoma cells **(E)**: CD163 positivity is remarkable in undifferentiated carcinoma cells **(F)**: Reactivity with CD33 in osteoclastic cells (long arrow) and tumor cells (short arrow) in pancreatic osteoclast-rich undifferentiated carcinoma.

In evolutionary terms, cancer cells can rapidly react and adapt to the changing tumor microenvironment in order to survive. In order for carcinoma cells to gain invasive characteristics, develop mobility, and metastasize, they must lose their epithelial properties (Figures 2A–C). This happens through the process of EMT, in which both the morphology and gene expression profiles of the cell change. Epithelial cells show mesenchymal cell characteristics after EMT [109]. The ability of cells to gain mobility through EMT and their ability to travel to distant tissues is also a very important molecular process in embryogenesis. In addition, cancers that undergo EMT change, possibly because of their stem cell properties, often develop resistance to chemotherapy and targeted therapies [109].

Many molecules in myeloid cells that take part in the microenvironment during the EMT process contribute to the EMT change directly or indirectly. As aforementioned, OCLs in bone metastases of breast carcinoma cause EMT in tumor cells through TGFβ, which is released during bone degradation [39, 40]. The morphological evidence for EMT and its requirement has been well established in preclinical and clinical studies [109, 110]. Nevertheless, recent studies have suggested that the EMT signature alone in breast cancer patients does not predict recurrence and disease-free survival [111, 112]. Dissemination via EMT conversion is a necessary limiting step in the metastatic process; however, it does not ensure successful colonization and outgrowth in secondary organs [113, 114]. We have described the effects of healthy myeloid cells and MDSCs, including OCLs, on the colonization, growth, and metastasis development of tumor cells in the metastatic focus. However, another effective mechanism in cancer progression and the development of metastatic ability is the mechanism of cell fusion (hybridization), in which myeloid cells are also involved.

**FIGURE 3:**
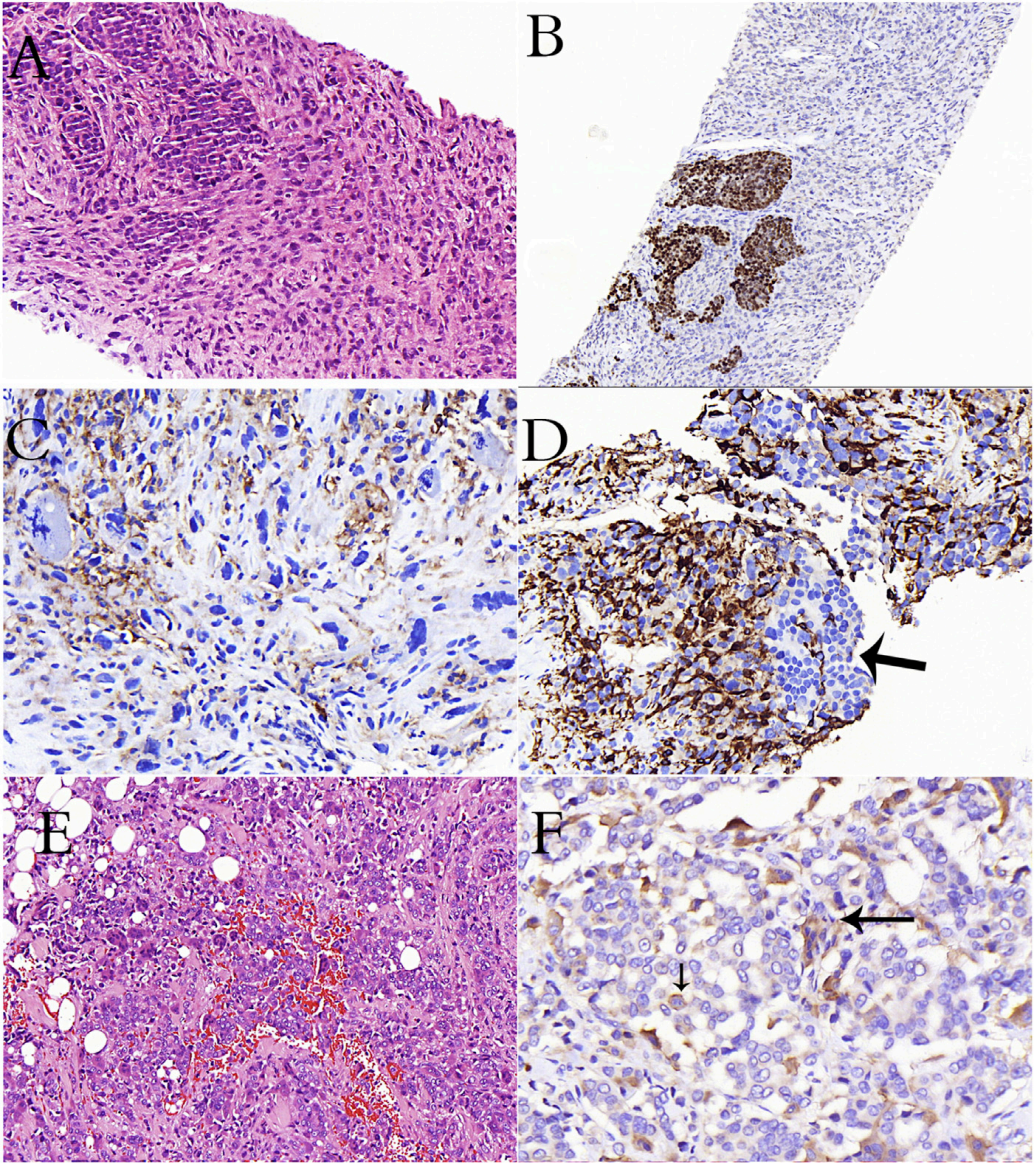
A case of breast carcinoma metastasized to the lymph node **(A–D)**. **(A)**: Epithelioid cancer cells and spindle-pleomorphic cells are intertwined (H&E stain). **(B)**: Estrogen positivity in epithelioid cells in immunohistochemical study **(C)**: Remarkable CD33 positivity in cells with pleomorphism and atypical mitosis in immunohistochemical study **(D)**: Remarkable CD163 positivity in pleomorphic cells, negativity in epithelioid cells (arrow) in immunohistochemical study **(E)**: Diffuse osteoclastic cells in a case of invasive breast carcinoma (H&E stain). **(F)**: CD33 reactivity in osteoclasts (long arrow) and cancer cells (short arrow) in immunohistochemical study.

While cell fusion is effective in physiological events such as fertilization, tissue regeneration, osteogenesis, embryogenesis, and the formation of multinuclear cells such as OCLs and trophoblasts, it also plays an active role in viral infection and cancer progression [115, 116]. Cancer cells can fuse with the endothelium, normal epithelial cells, and stromal cells. However, the most ideal cells for progression, invasion, and metastasis are myeloid cells and MSDCs. Under physiological conditions, OCLs, which are involved in bone remodeling, and monocytes from inflammatory cells are cells that can use fusion to form giant cells. In the fusion of cancer cells with myeloid cells, cancer cells may have motility, margination, transmigration, chemotaxis, phagocytosis, angiogenesis, matrix degradation, and resistance to chemotherapy of myeloid cells [105, 117, 118]. Additionally, they will also gain immunosuppressive properties against cancer via fusion with MDSCs. In breast cancer studies, it has been emphasized that the fusion of macrophages and cancer cells provides cancer cells with stem cell characteristics and reduces cancer cell apoptosis [117]. In multiple myeloma, it has been reported that the cells formed as a result of the fusion of myeloma cells and OCLs in the bone causes rapid bone degradation [119]. Mature myeloid cells (macrophages, OCLs, and granulocytes) and TAM cells are highly fusogenic cells. The densities of cells bearing M2 macrophage characteristics without fusion in the primary tumor microenvironment were found to be associated with breast cancer survival [120]. Since the hybrid cell that emerges at the end of the fusion of TAM cells with cancer cells has the characteristics of both myeloid and cancer cells, the cell is directly related to tumor aggression, metastasis, and prognosis [117, 120, 121]. In some patients with metastatic breast carcinoma, we observed stronger staining with CD33, CD168 and CD68 immunohistochemical stains, especially in spindle and pleomorphic cells compared to epithelioid cells (Figures 3A–D). Similarly, we observed diffuse positivity in osteoclast rich undifferentiated carcinomas (Figures 2D,E). We also observed staining with CD33 in the breast carcinoma case containing OCL (Figures 3E,F) and osteoclast-rich undifferentiated carcinoma cases (Figure 2F). This result suggests that osteoclastic cells may have developed primarily from immature monocytes (TAGs). We also detected undifferentiated carcinoma cells in osteoclast cells in our osteoclast-rich thyroid undifferentiated carcinoma case (Figures 1E,F). This may be due to the fusion of OCL with the cancer cell or its phagocytic feature.

In conclusion, myeloid cells contribute to tumorigenesis, tumor metastasis, and progression both in the primary tumor (especially M2 macrophages, MSDCs) and metastatic tumor microenvironment (OCLs and M2 macrophages, MSDCs). They also contribute to the formation of EMT, MET, and cancer stem cells. During cell fusion, hybrid cells formed by the fusion of cancer cells with macrophages, OCLs, and TAM cells also give cancer cells myeloid properties, causing tumor progression, metastasis, multinuclear giant cell formation, and cancer stem cell formation. Taking this into consideration, we believe that the concept of EMMT is more accurate for describing myeloid cells and cancer cells with myeloid properties than EMT.
